# Face and content validation of a novel aquablation training simulator: an educational project of the European school of urology

**DOI:** 10.1007/s11701-026-03388-4

**Published:** 2026-04-21

**Authors:** Engin Denizhan Demirkıran, Tarık Emre Şener, Davide Perri, Tiago Ribeiro de Oliveira, Juan Pablo Caballero, Luis Osorio, Ioannis Goumas Kartalas, Rodrigo Ramos, Afonso Sousa Castro, Daniel Oliveira Reis, Sergio Pereira, Laurian Dragos, Domenico Veneziano, Giorgio Bozzini, Bhaskar Somani, Evangelos Liatsikos

**Affiliations:** 1 Department of Urology, School of Medicine, Zonguldak Bülent Ecevit University, Zonguldak, Turkey; 2 Department of Urology, School of Medicine, Marmara University, Istanbul, Turkey; 3 Department of Urology, Azienda Socio Sanitaria Territoriale Lariana, Como, Italy; 4 Department of Urology, Armed Forces Hospital, Lisbon, Portugal; 5 Department of Urology, University General Hospital, Alicante, Spain; 6 Department of Urology, Hospital Lusíadas Porto, Porto, Portugal; 7 Department of Urology, Istituto Clinico Beato Matteo, Vigevano, Italy; 8 Department of Urology, Portuguese Institute of Oncology (IPO) Lisboa, Lisbon, Portugal; 9 Department of Urology, Hospital de Santo António. C.H.P. Porto, Porto, Portugal; 10 Department of Urology, Lisbon Medical Academic Centre, Lisbon, Portugal; 11 Department of Urology, Cambridge University Hospitals NHS Foundation Trust, Cambridge, UK; 12 Department of Urology, Bronxcare Health System, NY, USA; 13 Department of Urology, University Hospital Southampton NHS Foundation Trust, Southampton, UK; 14 Department of Urology, University Hospital Patras, Patras, Greece

**Keywords:** Aquablation, Simulation, Training, Validation, Endourology

## Abstract

Aquablation is an innovative robotic waterjet procedure designed to treat benign prostatic obstruction (BPO) by combining real-time transrectal ultrasound (TRUS) guidance with automated tissue removal using water jets. With its increasing adoption throughout Europe, there is a crucial need for structured and validated training materials. This research represents an opportunistic expert evaluation conducted within a European School of Urology (ESU) educational setting and provides preliminary validity evidence by assessing the face and content validity of an Aquablation training simulator, focusing on realism, procedural accuracy, and suitability for training. A cross-sectional validation study was conducted involving expert endourologists who are familiar with Aquablation. Participants completed standardized questionnaires to assess face and content validity, using 4-point Likert scales. Descriptive statistics were computed, along with measures of internal consistency (Cronbach’s α) and Content Validity Indices (I-CVI, S-CVI/Ave, S-CVI/UA). Face validation demonstrated a high degree of realism and strong consensus across all items, achieving a Cronbach’s alpha of 0.92. Content validation showed excellent agreement, with an S-CVI/Ave of 0.91 and an S-CVI/UA of 0.50. Experts unanimously supported the model’s integration into structured Aquablation training programs. The expert validation confirms the simulator’s educational relevance and procedural accuracy, supporting its use as a training platform for Aquablation within lower urinary tract endoscopy education. Next steps will include defining curriculum components guided by CTA/Delphi and establishing objective performance metrics; subsequent work will evaluate construct and predictive validity across multiple centers.

**Supplementary Information:**

The online version contains supplementary material available at 10.1007/s11701-026-03388-4.

## Introduction

Benign prostatic obstruction (BPO) remains one of the most prevalent urological conditions affecting aging men worldwide [1]. Although transurethral resection of the prostate (TURP) has traditionally been regarded as the surgical gold standard, it is linked with a learning curve, intraoperative bleeding, and postoperative morbidity [2]. In recent years, advancements in technology have resulted in the development of less invasive options designed to minimize perioperative complications while ensuring durable functional results [3]. 

Aquablation is among the most cutting-edge technologies available. This procedure utilizes a robotic system to perform automated, accurate, and consistent resection of prostate tissue by employing a high-speed saline waterjet, guided in real-time by transrectal ultrasound (TRUS). After identifying the ablation zones on integrated TRUS images, the robotic handpiece utilizes a high-pressure waterjet to accurately resect prostatic tissue while preserving critical anatomical structures like the verumontanum, external sphincter, and ejaculatory ducts. The procedure is generally conducted with direct endoscopic visualization, utilizing the waterjet for rapid tissue removal. Initial clinical findings have shown that Aquablation offers similar enhancements in urinary flow and symptom relief as TURP and enucleation procedures, but with notably shorter operation times and reduced risks of sexual dysfunction and urinary incontinence [4–6]. 

Despite its clinical promise, the restricted availability of clinical cases and the high expense associated with clinical implementation highlight the necessity for validated training platforms. Simulation-based training has become an essential part of contemporary surgical education, enabling the safe development of skills outside the operating room. Nonetheless, the educational value of these simulators relies on thorough validation to ensure that they faithfully replicate the procedural and anatomical aspects of surgery.

Aligned with contemporary validity frameworks in simulation-based education, this project was designed as a stepwise evaluation program: the present study reports an opportunistic expert evaluation of face and content validity and the station’s suitability for structured training. Next steps will include cognitive task analysis (CTA) and formal expert-consensus method using a Delphi approach to refine the task breakdown, training requirements, and assessment criteria; subsequent work will evaluate construct validity using objective performance metrics and explore skill transfer through multi-center implementation.

The Aquablation simulation model is designed to replicate the essential steps of aqua-ablative prostate surgery, such as TRUS-guided contouring, robotic planning, and waterjet ablation. Prior to its integration in structured training programs, this model requires a systematic assessment of its face and content validity. Importantly, the present study was not intended to define or replace a full ESU curriculum pathway. Rather, it signifies a preliminary educational assessment driven by needs, carried out during European Urology Residents Education Programme (EUREP) 2025, with the goal of evaluating assessment perceived realism and procedural relevance of an Aquablation simulation environment already in use. In the context of the ESU/ Standardisation in Surgical Education (SISE) framework, this study should be seen as an initial effort aimed at guiding future curriculum development, rather than as a completed training protocol. Accordingly, this work should be interpreted as a Phase 0 opportunistic expert appraisal of a prototype station delivered at a large-scale educational meeting, designed to capture early validity evidence and guide the subsequent ESU-standard curriculum steps.

## Materials and methods

A cross-sectional validation study was carried out to assess the face and content validity of the Aquablation model. The validation adhered to established frameworks for evaluating surgical simulators [7]. 

The evaluators were expert endourologists from different centers with substantial experience in the surgical treatment of BPH, particularly TURP and endoscopic enucleation. Prior familiarity with Aquablation varied among participants. All assessments were performed during a structured training session at EUREP 2025 in Prague, Czech Republic, ensuring a standardized evaluation setting, while a degree of selection cannot be excluded. Each member of the panel independently assessed the model and filled out standardized validation questionnaires following a demonstration and hands-on experience.

**Fig. 1:**
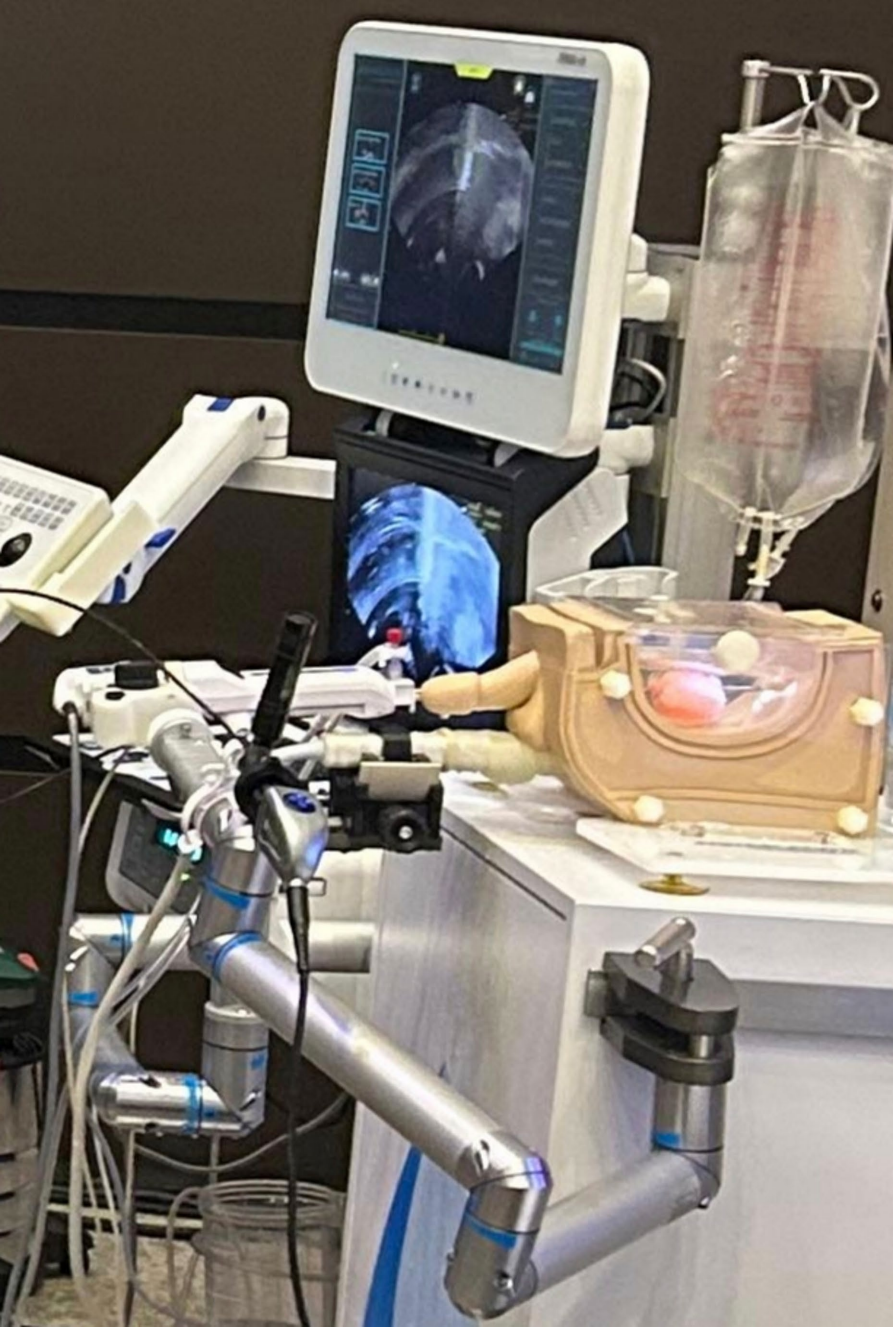
Aquablation training simulator setup with integrated imaging and phantom model

The AquaBeam^®^ Robotic System (PROCEPT BioRobotics, California, USA), EndoUroPhantom training box and 3D-printed prostate phantoms by Quantitative Surgical GmbH (Mannheim, Germany) are used in Aquablation procedures [8]. The model was specifically designed to replicate the procedural workflow of prostate surgery in a structured training environment. (Fig. 1)

The phantom model incorporates a synthetic prostate module integrated into a dedicated housing system adapted for Aquablation training. The prostate model was derived from patient imaging of a representative 60 cc prostate and is manufactured from soft, non-toxic biomimetic hydrogel materials designed to approximate the mechanical properties of prostatic tissue.

The simulator integrates three main components:

1. Anatomical representation of the prostate, bladder neck, and urethral landmarks to mimic realistic treatment planning and intraoperative orientation.
2. Ultrasound-based guidance system, replicating transrectal ultrasound (TRUS) views for prostate mapping, contouring, and ablation planning.
3. Software and waterjet ablation interface, simulating probe placement, contour definition, and real-time Aquablation with visual feedback of tissue clearance.

**Fig. 2:**
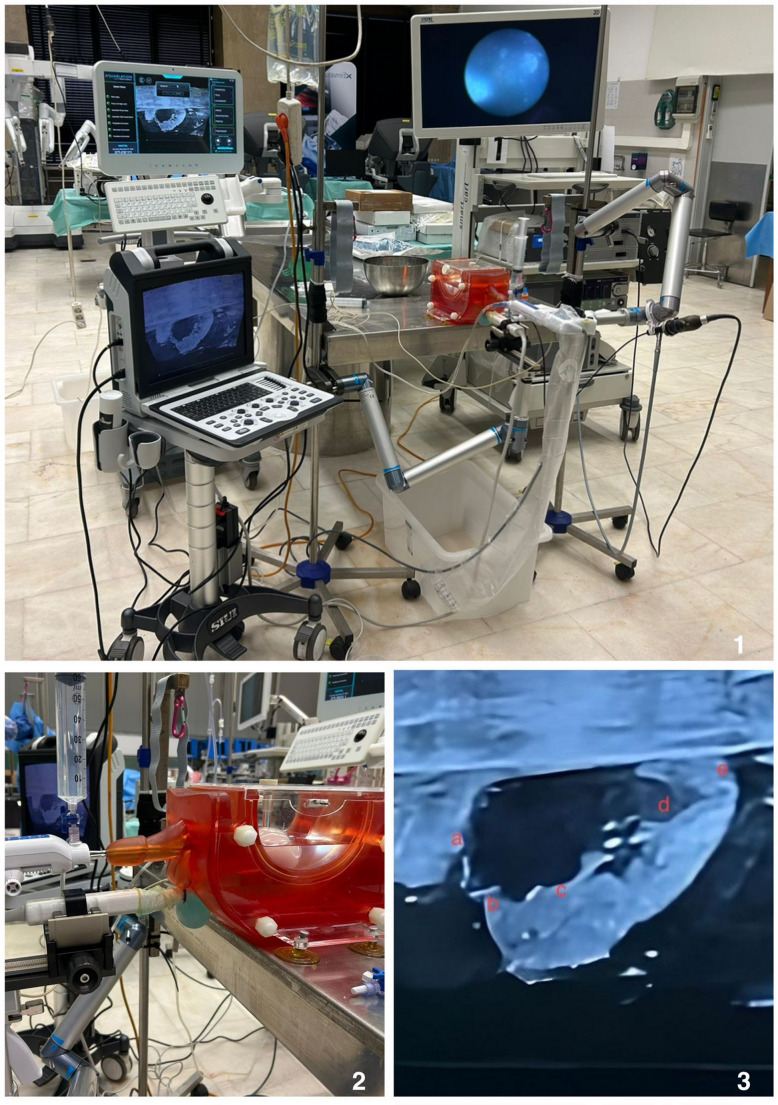
(**1**) Full simulation environment replicating clinical Aquablation workflow. (**2**) Close-up view of the training box and prostate phantom. (**3**) Ultrasound view of phantom prostate post aquablation procedure (a. Treatment start point, b. Bladder neck, c. Mid-prostate, d. Ejaculation sparing area, e. Treatment end point)

This approach provided a standardized and reproducible anatomical model for simulation-based training. (Fig. 2) (Suppl. Video).

The model does not reproduce bleeding control or fully realistic tissue behavior, as its primary purpose is to simulate the planning and robotic Aquablation phase rather than the subsequent endoscopic hemostatic step.

### Task deconstruction and questionnaire development

Before creating the survey, the Aquablation procedure was deconstructed into its fundamental steps by faculty members experienced in Aquablation and endourological training. This pragmatic task deconstruction focused on essential elements of the workflow and was organized into six domains: (1) prostate mapping with TRUS, (2) contour definition, (3) probe alignment, (4) waterjet ablation, (5) identification of procedural endpoints, and (6) basic troubleshooting. The resulting domain structure was used to generate an initial item pool reflecting the core Aquablation workflow as represented by the training station. This process was not a formal CTA and was not intended to define a complete ESU curriculum or proficiency benchmarks; rather, it supported Phase 1 expert appraisal of realism and perceived relevance, and it provides a framework for the planned CTA and Delphi-based consensus steps.

The questionnaires for face and content validation were subsequently designed based on these established procedural elements, focusing on an initial, needs-based educational assessment rather than employing a formal CTA or Delphi process.

The face and content validity questionnaires were developed based on previously published studies on surgical simulation validation and adapted to the specific procedural steps and educational objectives of Aquablation [9–12]. The items were prepared by a subgroup of experts from the ESU Lower Urinary Tract Endoscopy Working Group, informed by the existing literature. Not all evaluators participated in questionnaire development. The final versions were reviewed and approved by the ESU Lower Urinary Tract Endoscopy Working Group.

This study was intentionally limited to evaluating realism and content coverage through expert judgment using standardized questionnaires; it did not seek to finalize a curriculum derived from CTA or establish proficiency benchmarks.

An 11-item questionnaire was used for face validation to evaluate realism, anatomical precision, procedural representation, and the applicability for training. Each item was scored on a 4-point Likert scale. (1 = Strongly Disagree, 2 = Disagree, 3 = Agree, 4 = Strongly Agree). Similarly, for content validation, a 12-item questionnaire focused on relevance was used to assess key procedural aspects of Aquablation. This included TRUS guidance, prostate contouring, positioning the probe, performing the ablation, managing complications, and addressing troubleshooting. Each item was evaluated on a 4-point relevance scale. (1 = Not Relevant, 2 = Somewhat Relevant, 3 = Quite Relevant, 4 = Highly Relevant). A final dichotomous question inquired if the model should be suggested for integration into structured Aquablation training programs.

### Data analysis

Descriptive statistics and percentage agreement (≥ 3) were computed to evaluate face validity. Cronbach’s alpha was utilized to determine internal consistency. An analysis of item-total correlation was conducted to evaluate how each item contributed to the overall scale. Likert distribution plots were created to depict levels of agreement visually.

To assess content validity, the Item-Level Content Validity Index (I-CVI) was determined by calculating the proportion of experts who rated each item as either 3 or 4. The Scale-Level CVI/Average (S-CVI/Ave) was defined as the average of all I-CVI scores, while the S-CVI/Universal Agreement (S-CVI/UA) represented the percentage of items for which all evaluators gave a score of 3 or higher. An I-CVI of at least 0.78 and an S-CVI of at least 0.90 were deemed acceptable. Additionally, the percentage of experts who recommended incorporating the model into structured training programs was documented.

### Ethics

Some of the expert evaluators were also included as co-authors due to their contribution to the study design and interpretation. To minimize potential bias, all evaluations were performed independently, and responses were collected and analysed in an aggregated manner without financial or commercial incentives.

## Results

### Face validation

Twelve expert urologists participated in and completed both validation surveys. The face validity survey revealed a strong consensus among experts about the Aquablation model’s realism and its effectiveness for training. The descriptive analysis indicated that mean scores were above 3.4 for all items, with overall agreement rates surpassing 90%. Cronbach’s alpha was calculated at 0.92, reflecting excellent internal consistency. (Table 1)

Analysis of item-total correlations demonstrated that each item had a strong association with the overall score, ranging from 0.53 to 0.88, which indicates the survey instrument’s internal consistency. Likert scale distribution showed that most participants rated the model as Agree or Strongly Agree for all items, with very few expressing disagreement.

### Content validation

Expert evaluators also gave high marks to the content validity of the Aquablation model. The Scale-Level CVI (S-CVI/Ave) was 0.91, signifying excellent overall content validity, while the S-CVI/UA was 0.50, indicating that half of the items received unanimous agreement from the reviewers.

At the item level, the majority of skills and procedural steps exhibited I-CVI values exceeding 0.80, indicating a strong agreement on their importance. Average scores were consistently 3.5 or higher, with medians reaching 4 (Highly Relevant). (Table 2)

Although certain elements, such as the smooth transition between lobes and the novice–expert distinction, received slightly less agreement, the unanimous endorsement for its inclusion in the curriculum underscores its significance as a high-fidelity educational tool.

**Table 1:** Face Validation Results for the Aquablation Simulation Model

Item	Mean ± SD	Median	% Agreement (≥ 3)
Anatomical appearance of prostate lobes and bladder neck is realistic	3.25 ± 0.75	3.0	83.3%
Ultrasound imaging reflects real TRUS views	3.75 ± 0.45	4.0	100%
Targeting/contouring interface mimics AquaBeam software	3.75 ± 0.45	4.0	100%
Waterjet ablation simulates real tissue interaction	3.67 ± 0.49	4.0	100%
Visualization challenges (e.g., poor contrast zones) are realistic	3.42 ± 0.90	4.0	91.7%
Anatomical landmarks (verumontanum, urethra) positioned accurately	3.50 ± 0.52	3.5	100%
Response of model to ablation (tissue clearance) is realistic	3.67 ± 0.49	4.0	100%
Sequence of steps reflects actual Aquablation procedures	3.75 ± 0.45	4.0	100%
Overall simulation realistic for aquaablative surgery	3.67 ± 0.49	4.0	100%
Suitable for training basic Aquablation skills	3.67 ± 0.49	4.0	100%
Suitable for evaluating Aquablation competency	3.58 ± 0.51	4.0	100%

**Table 2:** Content Validation Results for the Aquablation Simulation Model

Item	Mean ± SD	Median	I-CVI
Correct identification of bladder neck and verumontanum using TRUS guidance	3.42 ± 0.90	4.0	0.75
Accurate prostate mapping and contour definition via software interface	3.67 ± 0.49	4.0	1.00
Proper probe placement and angle alignment within the urethra	3.75 ± 0.45	4.0	1.00
Adjustment of ablation zone to spare critical areas (e.g., verumontanum, sphincter)	3.75 ± 0.45	4.0	1.00
Execution of ablation in a smooth and continuous manner without deviation	3.50 ± 0.52	3.5	1.00
Recognition and management of complications (e.g., incomplete ablation, perforation)	3.08 ± 1.00	3.0	0.75
Transition between anterior and posterior lobe treatment smoothly	3.17 ± 0.94	3.0	0.83
Post-procedure evaluation of tissue removal and cavity shape	3.50 ± 0.80	4.0	0.83
Troubleshooting software or device-related issues during the procedure	3.50 ± 0.67	4.0	0.92
Overall, the model covers the critical steps necessary for learning Aquablation	3.83 ± 0.39	4.0	1.00
The model is appropriate for distinguishing between novice and advanced learners	3.25 ± 0.97	3.5	0.83
The model is useful for Aquablation training overall	3.75 ± 0.45	4.0	1.00

## Discussion

The research revealed that the newly developed Aquablation simulator demonstrated outstanding face and content validity, affirming its authenticity, procedural accuracy, and appropriateness for structured training in aqua-ablative prostate surgery. Experts gave the simulator high ratings in most areas, with internal consistency being excellent (Cronbach’s α = 0.92). The strong average ratings and high percentage of agreement found in both face and content validation underscore the simulator’s educational significance. It is crucial to understand that these results serve as initial validity evidence for the incorporation of the station into structured teaching. However, they do not substitute for the CTA/Delphi-driven curriculum specification and metric development necessary for proficiency-based training and establishing construct validity.

According to the face validity results, experts gave the simulator high marks for its anatomical accuracy, the endoscopic setting, and its overall effectiveness for training. The model was especially appreciated for allowing practice of essential procedural steps such as the anatomical depiction of organs, TRUS views for prostate mapping, contouring, and planning for ablation. It also included features like the waterjet ablation interface, simulating probe placement, defining contours, and real-time Aquablation with visual feedback on tissue removal. Engaging in repetitive exercises within controlled environments has been demonstrated to boost skill levels, with research showing that individuals who consistently practice with simulations achieve notably better results in real surgical procedures [13, 14]. The results indicate that the simulator is highly effective for initial training stages, enabling residents to build confidence in the fundamental technical aspects of Aquablation without any patient-related risk. Nevertheless, the simulation does not encompass the replication of intraoperative difficulties, such as bleeding and variable tissue resistance, which the model is unable to demonstrate. This underscores a fundamental limitation that should be considered when determining its place in a comprehensive training program. However, an advantage of the training model is that the procedure can be directly observed with the naked eye, thanks to the transparent training box in which it is performed.

Simulation-based training has become a crucial part of contemporary urological education, offering a secure and standardized setting for developing skills without carrying the patient-related risks. Simulation fidelity is related to the extent to which a simulator mimics real-world situations, including both its physical look and operational features. Although conventional definitions emphasize technological advancements and visual details, studies have indicated that the link between fidelity and learning outcomes is complex [15]. 

Previous validation research on transurethral and endoscopic simulators, such as those created for TURP, HoLEP, and TURB, has consistently highlighted the significance of anatomical accuracy and task fidelity for effective learning. In line with these models, the Aquablation simulator exhibited high face validity, with experts strongly affirming its realism and accurate representation of procedures [16–18]. 

In contrast to traditional endoscopic methods, Aquablation is fundamentally distinct because it incorporates real-time TRUS guidance and robotic planning, elements that are challenging to replicate with standard bench models. The high scores for ultrasound fidelity and the accuracy of the targeting interface in this study indicate that the simulator effectively mimics these unique procedural features. This makes the model a valuable educational resource, bridging the gap between theoretical knowledge of robotic waterjet ablation and the development of practical skills.

The educational relevance of the present simulator is further supported by the work of Faber et al., who demonstrated the feasibility and accuracy of image-guided, robot-assisted prostate ablation using high-velocity waterjet hydrodissection in a preclinical setting [19]. Their research demonstrated successful tissue removal while preserving critical anatomical structures using real-time TRUS guidance, which serves as the technological basis for contemporary Aquablation surgery. The simulator evaluated in this study replicates fundamental principles -particularly TRUS-guided contouring and automated waterjet execution- thus transforming initial technological validation into a well-organized educational platform ideal for standardized training.

The results indicate that the simulator can be effectively integrated as a validated training station into structured programs for basic Aquablation training if supported by CTA and expert consensus, such as those led by the ESU, which include structured training and competency evaluation. Learning through simulation has been proven to improve psychomotor skills and shorten the learning curve prior to exposure in the operating room [20]. Considering the expense and limited clinical availability of the AquaBeam^®^ system, validated simulators could enable wider access to training and assist in standardizing technique acquisition across various centers. This is consistent with research that supports the use of structured educational programs that incorporates affordable and high-quality simulators to enhance surgical training in the field of urology [21, 22]. 

In the context of the ESU/SISE educational framework, the simulator assessed in this study should be classified as a Lower Urinary Tract Step 2 training tool [23]. Its primary purpose is to aid in skill development, familiarization, and initial procedural comprehension. At this point, it is not designed to serve as a platform for proficiency-based training or assessment.

### Limitations

Several limitations should be acknowledged. To begin with, the number of expert raters was limited. Additionally, this manuscript presents an initial validation study, which focuses on face and content validity, representing the earliest levels of validation in simulation-based research, relying on expert opinions rather than objective performance metrics. Also, this study did not incorporate a formal CTA or a Delphi consensus process, both of which are regarded as crucial elements in the comprehensive development of the ESU/SISE curriculum. Therefore, the simulator design and evaluation were based on expert experience rather than on a standardized, consensus-based procedural framework. Although a structured needs assessment could have provided additional educational context, it would not replace the value of a formal CTA and Delphi process. As a result, construct validity and objective performance measures were not evaluated but are planned to be completed in subsequent studies.

This limitation was intentional, as the main aim of this study was to offer initial evidence of face and content validity for a simulator already employed in practical training environments. The next steps, which include defining the curriculum through CTA/Delphi methods, selecting metrics, and testing construct validity, are planned and necessary before the simulator can be considered as a training and evaluation tool for the finalized ESU training protocol as previous ones [24]. 

Future studies should focus on using objective performance evaluation. The ESU working group is set to perform the CTA and Delphi consensus, to outline the Aquablation task division, necessary skills, and evaluation criteria, such as identifying TRUS landmarks, ensuring contour precision, maintaining safety margins, recognizing planning mistakes, and implementing troubleshooting measures. In the subsequent phase, the focus will be on assessing construct validity by examining objective outcomes such as time, accuracy, error rates, and specific critical errors. Additionally, the station will be tested in multi-center courses to investigate how skills are acquired and transferred to clinical settings.

## Conclusion

The Aquablation simulation model demonstrated promising face and content validity, with favorable expert ratings on realism, procedural accuracy, and educational significance. These findings support its potential inclusion in structured training programs within the developing field of robotic waterjet prostate surgery, laying the groundwork for advanced research into skill transfer and learning outcomes in endourological education. Further studies are required to establish construct validity and objective performance metrics.

## Supplementary Information

Below is the link to the electronic supplementary material.

Supplementary Material 1

## Data Availability

No datasets were generated or analysed during the current study.
